# 2-[(4-Formyl­phen­yl)(hy­droxy)meth­yl]acrylonitrile

**DOI:** 10.1107/S160053681102976X

**Published:** 2011-07-30

**Authors:** C. M. Sai Prasanna, K. Sethusankar, R. Rajesh, R. Raghunathan

**Affiliations:** a Department of Physics, Ethiraj College for Women, Chennai 600 008, India; b Department of Physics, RKM Vivekananda College (Autonomous), Chennai 600 004, India; c Department of Organic Chemistry, University of Madras, Guindy Campus, Chennai 600 025, India

## Abstract

In the title compound, C_11_H_9_NO_2_, the mean planes formed by the phenyl and acryl group are almost orthogonal to each other, with a dihedral angle of 88.61 (7)°. The carbonitrile side chain is almost linear, the C—C—N angle being 179.54 (16)°. In the crystal, mol­ecules are linked by inter­molecular O—H⋯O inter­actions into infinite chains running parallel to the *b* axis.

## Related literature

For uses of acrylonitrile derivatives, see: Ohsumi *et al.* (1998 ▶). For related structures, see: Cobo *et al.* (2005 ▶); Nizam Mohideen *et al.* (2007 ▶).
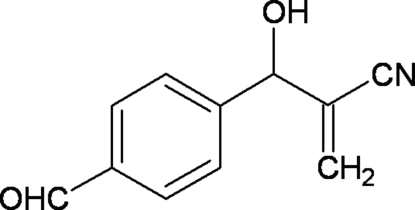


## Experimental

### 

#### Crystal data

C_11_H_9_NO_2_
*M* *_r_* = 187.19
Monoclinic, 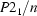

*a* = 7.6089 (5) Å
*b* = 6.0895 (3) Å
*c* = 20.5135 (14) Å
β = 93.615 (2)°
*V* = 948.59 (10) Å^3^
*Z* = 4
Mo *K*α radiation
μ = 0.09 mm^−1^
*T* = 293 K
0.30 × 0.20 × 0.20 mm

#### Data collection

Bruker Kappa APEXII CCD diffractometer
12108 measured reflections
2778 independent reflections
2109 reflections with *I* > 2σ(*I*)
*R*_int_ = 0.025

#### Refinement

*R*[*F* ^2^ > 2σ(*F* ^2^)] = 0.045
*wR*(*F* ^2^) = 0.137
*S* = 1.04
2778 reflections
128 parameters
H-atom parameters constrained
Δρ_max_ = 0.20 e Å^−3^
Δρ_min_ = −0.21 e Å^−3^

### 

Data collection: *APEX2* (Bruker, 2004 ▶); cell refinement: *SAINT* (Bruker, 2004 ▶); data reduction: *SAINT*; program(s) used to solve structure: *SHELXS97* (Sheldrick, 2008 ▶); program(s) used to refine structure: *SHELXL97* (Sheldrick, 2008 ▶); molecular graphics: *ORTEP-3* (Farrugia, 1997 ▶); software used to prepare material for publication: *SHELXL97* and *PLATON* (Spek, 2009 ▶).

## Supplementary Material

Crystal structure: contains datablock(s) global, I. DOI: 10.1107/S160053681102976X/pv2428sup1.cif

Structure factors: contains datablock(s) I. DOI: 10.1107/S160053681102976X/pv2428Isup2.hkl

Supplementary material file. DOI: 10.1107/S160053681102976X/pv2428Isup3.cml

Additional supplementary materials:  crystallographic information; 3D view; checkCIF report

## Figures and Tables

**Table 1:** Hydrogen-bond geometry (Å, °)

*D*—H⋯*A*	*D*—H	H⋯*A*	*D*⋯*A*	*D*—H⋯*A*
O2—H2⋯O1^i^	0.82	1.99	2.8107 (15)	175

Symmetry code: (i) 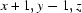.

**Fig. 1.**
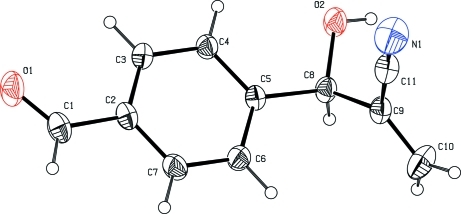
The molecular structure of the title compound showing 30% probability displacement ellipsoids.

**Fig. 2.**
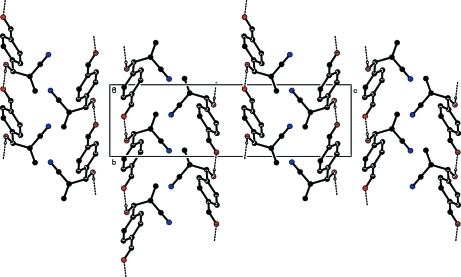
A view of the unit cell of the title compound viewed down a-axis; O—H···O intermolecular interactions are indicated by dashed lines.

## supplementary crystallographic information

### Comment

Acrylonitrile derivatives have been shown to possess antitubercular and antitumour activities (Ohsumi *et al.*, 1998).

In the title compound (Fig. 1), the mean planes formed by the phenyl ring C2—C7 and acryl group (N1/C8—C11) are almost orthogonal to each other with a dihedral angle 88.61 (7)°. The bond length  C9—C11 [1.4338 (18) Å] is significantly shorter than the expected value for a C—C single bond because of conjugation effects (Nizam Mohideen *et al.*, 2007). The mean plane of C2—C1—O1 is slightly twisted out of the mean plane of phenyl ring C2—C7 with a dihedral angle 2.62 (9)°. The carbonitrile side chain (C9—C11—N1) is almost linear, with the angle around central carbon atom being 179.54 (16)°. The title compound exhibits structural similarities with closely related structures (Cobo *et al.*2005, Nizam Mohideen *et al.*2007).

In the title compound, the crystal packing is stabilized by O2—H2···O1 intermolecular interactions which link the molecules into infinite chains running parallel to the *b*-axis (Tab. 1 & Fig. 2).

### Experimental

To a reaction mixture of terephthalaldehyde (1 mmol) and acrylonitrile (2 mmol) was added a catalytic quantity of 1,4-diazabicyclo[2.2.2]octane (10–15 mol %). The reaction mixture was left standing at room temperature in a stoppered sample flask. The progress of the reaction was monitored by Thin Layer Chromatography (TLC) over a period of several days. After 6 days the TLC revealed the presence of a product. The reaction mixture was dissolved in ethyl acetate and washed with aqueous HCl solution (0.25 *M*) and water followed by brine solution. The organic layer was separated and dried over sodium sulfate, filtering and evaporation of the organic solvent under reduced pressure. The product was seperated by flash column chromatography using hexane and ethyl acetate (4:1) as an eluent to give colorless solid. The product was dissolved in chloroform and heated for two minutes. The resulting solution was subjected to crystallization by slow evaporation of the solvent resulting in single crystals suitable for XRD studies.

### Refinement

The hydrogen atoms were placed in calculated positions with C—H = 0.93 to 0.98 Å and O—H = 0.82 Å and refined in the riding model with isotropic displacement parameters: *U*_iso_(H) = 1.2*U*_eq_(C) or 1.5*U*_eq_(O).

### Crystal data

**Table:**

C_11_H_9_NO_2_	*F*(000) = 392
*M**_r_* = 187.19	*D*_x_ = 1.311 Mg m^−^^3^
Monoclinic, *P*2_1_/*n*	Mo *K*α radiation, λ = 0.71073 Å
Hall symbol: -P 2yn	Cell parameters from 2778 reflections
*a* = 7.6089 (5) Å	θ = 2.0–30.1°
*b* = 6.0895 (3) Å	µ = 0.09 mm^−^^1^
*c* = 20.5135 (14) Å	*T* = 293 K
β = 93.615 (2)°	Block, colourless
*V* = 948.59 (10) Å^3^	0.30 × 0.20 × 0.20 mm
*Z* = 4	

### Data collection

**Table:**

Bruker Kappa APEXII CCD diffractometer	2109 reflections with *I* > 2σ(*I*)
Radiation source: fine-focus sealed tube	*R*_int_ = 0.025
graphite	θ_max_ = 30.1°, θ_min_ = 2.0°
ω scans	*h* = −10→10
12108 measured reflections	*k* = −8→5
2778 independent reflections	*l* = −28→28

### Refinement

**Table:**

Refinement on *F*^2^	Primary atom site location: structure-invariant direct methods
Least-squares matrix: full	Secondary atom site location: difference Fourier map
*R*[*F*^2^ > 2σ(*F*^2^)] = 0.045	Hydrogen site location: inferred from neighbouring sites
*wR*(*F*^2^) = 0.137	H-atom parameters constrained
*S* = 1.04	*w* = 1/[σ^2^(*F*_o_^2^) + (0.068*P*)^2^ + 0.1498*P*] where *P* = (*F*_o_^2^ + 2*F*_c_^2^)/3
2778 reflections	(Δ/σ)_max_ < 0.001
128 parameters	Δρ_max_ = 0.20 e Å^−^^3^
0 restraints	Δρ_min_ = −0.21 e Å^−^^3^

### Special details

**Table:**

Geometry. All e.s.d.'s (except the e.s.d. in the dihedral angle between two l.s. planes) are estimated using the full covariance matrix. The cell e.s.d.'s are taken into account individually in the estimation of e.s.d.'s in distances, angles and torsion angles; correlations between e.s.d.'s in cell parameters are only used when they are defined by crystal symmetry. An approximate (isotropic) treatment of cell e.s.d.'s is used for estimating e.s.d.'s involving l.s. planes.
Refinement. Refinement of *F*^2^ against ALL reflections. The weighted *R*-factor *wR* and goodness of fit *S* are based on *F*^2^, conventional *R*-factors *R* are based on *F*, with *F* set to zero for negative *F*^2^. The threshold expression of *F*^2^ > σ(*F*^2^) is used only for calculating *R*-factors(gt) *etc*. and is not relevant to the choice of reflections for refinement. *R*-factors based on *F*^2^ are statistically about twice as large as those based on *F*, and *R*- factors based on ALL data will be even larger.

### Fractional atomic coordinates and isotropic or equivalent isotropic displacement parameters (Å^2^)

**Table:**

	*x*	*y*	*z*	*U*_iso_*/*U*_eq_	
C1	0.29788 (17)	1.2762 (2)	0.08969 (7)	0.0530 (3)	
H1	0.2060	1.2372	0.1152	0.064*	
C2	0.45120 (15)	1.1299 (2)	0.09117 (6)	0.0402 (3)	
C3	0.59636 (15)	1.17738 (19)	0.05636 (6)	0.0403 (3)	
H3	0.5982	1.3042	0.0312	0.048*	
C4	0.73929 (15)	1.03554 (19)	0.05910 (6)	0.0377 (3)	
H4	0.8374	1.0683	0.0361	0.045*	
C5	0.73630 (14)	0.84482 (18)	0.09612 (5)	0.0341 (2)	
C6	0.58917 (16)	0.7961 (2)	0.12973 (6)	0.0440 (3)	
H6	0.5855	0.6670	0.1538	0.053*	
C7	0.44760 (16)	0.9386 (2)	0.12758 (6)	0.0473 (3)	
H7	0.3496	0.9059	0.1507	0.057*	
C8	0.88910 (15)	0.68441 (19)	0.09921 (6)	0.0386 (3)	
H8	0.8530	0.5530	0.0742	0.046*	
C9	0.94025 (16)	0.6160 (2)	0.16879 (6)	0.0416 (3)	
C10	0.9246 (2)	0.4136 (2)	0.19036 (8)	0.0658 (4)	
H10A	0.9592	0.3799	0.2335	0.079*	
H10B	0.8788	0.3045	0.1625	0.079*	
C11	1.01071 (18)	0.7847 (2)	0.21163 (7)	0.0499 (3)	
N1	1.0657 (2)	0.9196 (3)	0.24571 (8)	0.0783 (4)	
O1	0.28115 (14)	1.44271 (18)	0.05813 (6)	0.0647 (3)	
O2	1.03273 (12)	0.77961 (17)	0.07008 (5)	0.0550 (3)	
H2	1.1103	0.6874	0.0670	0.083*	

### Atomic displacement parameters (Å^2^)

**Table:**

	*U*^11^	*U*^22^	*U*^33^	*U*^12^	*U*^13^	*U*^23^
C1	0.0386 (6)	0.0578 (8)	0.0626 (8)	0.0154 (6)	0.0044 (6)	−0.0036 (6)
C2	0.0328 (5)	0.0454 (6)	0.0420 (6)	0.0090 (4)	−0.0012 (4)	−0.0055 (5)
C3	0.0394 (6)	0.0378 (6)	0.0434 (6)	0.0073 (4)	−0.0005 (5)	0.0030 (5)
C4	0.0336 (5)	0.0409 (6)	0.0390 (6)	0.0055 (4)	0.0047 (4)	0.0040 (4)
C5	0.0326 (5)	0.0371 (5)	0.0326 (5)	0.0067 (4)	0.0007 (4)	−0.0005 (4)
C6	0.0402 (6)	0.0457 (6)	0.0466 (7)	0.0051 (5)	0.0073 (5)	0.0104 (5)
C7	0.0337 (6)	0.0579 (7)	0.0512 (7)	0.0058 (5)	0.0099 (5)	0.0057 (6)
C8	0.0390 (6)	0.0381 (5)	0.0389 (6)	0.0101 (4)	0.0055 (4)	0.0029 (4)
C9	0.0417 (6)	0.0405 (6)	0.0426 (6)	0.0102 (5)	0.0018 (5)	0.0033 (5)
C10	0.0954 (12)	0.0465 (8)	0.0535 (8)	0.0048 (8)	−0.0100 (8)	0.0109 (6)
C11	0.0546 (7)	0.0469 (7)	0.0476 (7)	0.0105 (6)	−0.0009 (6)	0.0022 (5)
N1	0.0984 (12)	0.0638 (8)	0.0706 (9)	0.0003 (8)	−0.0120 (8)	−0.0121 (7)
O1	0.0556 (6)	0.0605 (6)	0.0781 (7)	0.0279 (5)	0.0057 (5)	0.0043 (5)
O2	0.0419 (5)	0.0599 (6)	0.0653 (6)	0.0204 (4)	0.0195 (4)	0.0194 (5)

### Geometric parameters (Å, °)

**Table:**

C1—O1	1.2055 (18)	C6—H6	0.9300
C1—C2	1.4664 (16)	C7—H7	0.9300
C1—H1	0.9300	C8—O2	1.4036 (15)
C2—C3	1.3829 (17)	C8—C9	1.5139 (16)
C2—C7	1.3853 (18)	C8—H8	0.9800
C3—C4	1.3871 (15)	C9—C10	1.3173 (18)
C3—H3	0.9300	C9—C11	1.4338 (18)
C4—C5	1.3887 (15)	C10—H10A	0.9300
C4—H4	0.9300	C10—H10B	0.9300
C5—C6	1.3835 (16)	C11—N1	1.1410 (19)
C5—C8	1.5168 (14)	O2—H2	0.8200
C6—C7	1.3818 (17)		
			
O1—C1—C2	125.37 (14)	C6—C7—C2	120.24 (11)
O1—C1—H1	117.3	C6—C7—H7	119.9
C2—C1—H1	117.3	C2—C7—H7	119.9
C3—C2—C7	119.84 (10)	O2—C8—C9	110.75 (10)
C3—C2—C1	121.51 (12)	O2—C8—C5	109.36 (9)
C7—C2—C1	118.64 (12)	C9—C8—C5	111.56 (9)
C2—C3—C4	119.92 (11)	O2—C8—H8	108.4
C2—C3—H3	120.0	C9—C8—H8	108.4
C4—C3—H3	120.0	C5—C8—H8	108.4
C3—C4—C5	120.20 (11)	C10—C9—C11	120.17 (13)
C3—C4—H4	119.9	C10—C9—C8	123.43 (12)
C5—C4—H4	119.9	C11—C9—C8	116.39 (11)
C6—C5—C4	119.58 (10)	C9—C10—H10A	120.0
C6—C5—C8	118.90 (10)	C9—C10—H10B	120.0
C4—C5—C8	121.50 (10)	H10A—C10—H10B	120.0
C7—C6—C5	120.20 (11)	N1—C11—C9	179.54 (16)
C7—C6—H6	119.9	C8—O2—H2	109.5
C5—C6—H6	119.9		
			
O1—C1—C2—C3	1.9 (2)	C3—C2—C7—C6	0.60 (19)
O1—C1—C2—C7	−177.02 (14)	C1—C2—C7—C6	179.52 (12)
C7—C2—C3—C4	−1.32 (18)	C6—C5—C8—O2	−171.48 (11)
C1—C2—C3—C4	179.80 (11)	C4—C5—C8—O2	10.23 (15)
C2—C3—C4—C5	0.69 (18)	C6—C5—C8—C9	−48.61 (15)
C3—C4—C5—C6	0.66 (17)	C4—C5—C8—C9	133.09 (11)
C3—C4—C5—C8	178.94 (10)	O2—C8—C9—C10	−122.89 (15)
C4—C5—C6—C7	−1.38 (19)	C5—C8—C9—C10	115.05 (15)
C8—C5—C6—C7	−179.71 (11)	O2—C8—C9—C11	56.40 (14)
C5—C6—C7—C2	0.8 (2)	C5—C8—C9—C11	−65.67 (14)

### Hydrogen-bond geometry (Å, °)

**Table:**

*D*—H···*A*	*D*—H	H···*A*	*D*···*A*	*D*—H···*A*
O2—H2···O1^i^	0.82	1.99	2.8107 (15)	175

Symmetry codes: (i) *x*+1, *y*−1, *z*.
